# Comparisons of Risk Factors for Abdominal Aortic Aneurysm and Coronary Heart Disease: A Prospective Cohort Study

**DOI:** 10.1177/0003319720946976

**Published:** 2020-08-07

**Authors:** Jun Xiao, Yan Borné, Xue Bao, Margaretha Persson, Anders Gottsäter, Stefan Acosta, Gunnar Engström

**Affiliations:** 1 Department of Cardiovascular Surgery, 5193 Fujian Medical University Union Hospital, Fuzhou, China; 2 Department of Clinical Sciences, Malmö, 5193 Lund University, Malmö, Sweden; 3 Department of Cardiology, 66506 Nanjing Drum Tower Hospital Clinical College of Nanjing Medical University, Nanjing, China; 4 Vascular Centre, Department of Cardiothoracic and Vascular Surgery, 5193 Skåne University Hospital, Malmö, Sweden

**Keywords:** abdominal aortic aneurysm, coronary heart disease, proportional hazards models, smoking, diabetes mellitus

## Abstract

Even though abdominal aortic aneurysm (AAA) and coronary heart disease (CHD) are both related to atherosclerosis, there could be important differences in risk factors. Based on Malmö Diet and Cancer Cohort, the incidence of AAA and CHD was followed prospectively. Cox regression was used to calculate the association of each factor with AAA and CHD and hazards ratio were compared using a modified Lunn-McNeil method; 447 participants developed AAA and 3129 developed CHD. After multivariate adjustments, smoking, antihypertensive medications, lipid-lowing medications, systolic and diastolic blood pressures, apolipoprotein (Apo) A1 (inversely), ApoB, ApoB/ApoA1 ratio, total leukocyte count, neutrophil count, and neutrophil to lymphocyte ratio were associated with the risks of both AAA and CHD. When comparing risk factor profiles for the 2 diseases, smoking, diastolic blood pressure, ApoA1, and ApoB/ApoA1 ratio had stronger associations with risk of AAA than with risk of CHD, while diabetes and unmarried status showed increased risk of CHD, but not of AAA (all *P* values for equal association <.01). The results from this big population study confirm that the risk factor profiles for AAA and CHD show not only many similarities but also several important differences.

## Introduction

Abdominal aortic aneurysms (AAAs) are a significant health burden, particularly among elderly males with an estimate of 1.3% of deaths being caused by AAA in men aged 65 to 85 years in developed countries.^1^ Abdominal aortic aneurysms seldom cause obvious symptoms, except at rupture, when it is often fatal.^2^ Thus, it is important to identify individuals at high risk of AAA. Coronary heart disease (CHD) and AAA share many cardiovascular (CV) risk factors and CHD is a common comorbidity in AAA, and vice versa.^3–5^

A meta-analysis including 23 studies reported a positive association between CHD and the occurrence of subclinical AAA and showed that CHD is a strong predictor of future AAA events.^6^ Both AAA and CHD are considered to be different manifestations of atherosclerosis. Epidemiological studies indicate that AAA and CHD share some common risk factors, such as age, male gender, hypertension, dyslipidemia, and smoking, which are promotors of atherosclerosis.^6–8^ Moreover, inflammatory responses in the aortic walls were observed in both diseases.^9^

However, the definite pathogenesis of AAA is still unclear and the relation between atherosclerosis and AAA has recently been increasingly challenged.^7,10^ Some atherosclerotic risk factors, such as hypercholesterolemia and obesity, are not pronounced risk factors for AAA.^8^ Diabetes mellitus (DM) is definitely a risk factor for CHD, whereas it seems to reduce the risk for AAA.^11,12^ Also, the relationship with inflammatory biomarkers have varied between 2 diseases, and some biomarkers, such as interferon-γ, interleukin-5, and interleukin-6, have even shown opposite relationships.^8,9^

Studies of CV risk factors and their relationships with atherosclerotic diseases in different vascular beds could improve our understanding of atherogenesis. We aimed to compare the risk factor profiles of AAA and CHD and identify their shared and specific risk factors in a Swedish population-based cohort study, and formally test the differential relationship regarding the incidence of AAA and CHD.

## Methods

### Participants

The Malmö Diet and Cancer study (MDCS) is a prospective cohort study, recruiting participants from the general population in the city of Malmö, Sweden. The baseline information was collected between March 1991 and September 1996 among 12 120 males and 18 326 females, including peripheral venous blood samples, physical examination, and a self-administered questionnaire. Of the initial 30 446 participants, complete information on covariates and outcome was available for 27 246 participants (Supplementary Figure 1). Twenty-one patients with total leukocyte count higher than 20 × 10^9^ /L were excluded to rule out acute inflammation or laboratory errors. We further excluded 23 patients with previous AAA and 514 patients with previous CHD. The final study population consisted of 26 688 participants (10 262 males and 16 426 females, aged 45-73 years). There were 3168 excluded patients with missing baseline covariates and without history of AAA and CHD. Among the 3168 excluded patients, 46 individuals had AAA during follow-up (0.79 per 1000 person-years) and 438 had CHD (7.83 per 1000 person-years). In the final study population (n = 26 688), the incidence rates of AAA and CHD were 0.85 and 6.08 per 1000 person-years, respectively.

The study was approved by the regional ethics committee (LU 51/90). All procedures performed in this study were in accordance with the ethical standards of the 1964 Helsinki Declaration and its later amendments. Informed consent was obtained from all participants.

### Baseline Information

Data on marital status, education level, smoking habits, use of antihypertensive, and lipid-lowing medications were derived from a self-administered questionnaire. Marital status was modeled as married, single, divorced, or widow/widower. Education level was categorized as school years into low (<9), median (9-12 years), and high (>12 years). Smoking was assessed in the questionnaire. The question was “Do you smoke?” and there were 4 response alternatives: 1 = Yes, I smoke regularly; 2 = Yes, I smoke occasionally; 3 = No, I have stopped smoking; and 4 = No, I have never smoked. Waist circumference was measured in the midpoint between the iliac crest and the lowest rib. After 10 minutes of rest in the supine position, blood pressure (BP) was measured using a mercury-column sphygmomanometer. Diabetes mellitus was defined as a self-reported physician’s diagnosis, or self-reported use of antidiabetic medications, or a diagnosis of DM from national or regional hospital registers.^13^

### Laboratory Measurements

Blood samples were drawn from an antecubital vein. Blood cell counts were measured in fresh blood, according to standard procedures at the Department of Clinical Chemistry, Malmö University Hospital. The measurements of total leukocyte count and counts of leukocyte subtypes, including neutrophils, lymphocytes, and a group of mixed cell types (monocytes, eosinophils, and basophils), have been described.^14^ Apolipoproteins A1 (ApoA1) and B (ApoB) were measured at Quest Diagnostics, using an immunonephelometric assay run on a Siemens BNII. The interassay variability was <4.0% for both ApoA1 and ApoB. The ApoB/ApoA1 ratio was calculated as the ratio of ApoB/ApoA1 measured in the same blood sample.

### Ascertainment of Outcomes and Validation

Participants were followed from baseline examination until death, migration from Sweden, end of follow-up (December 31, 2016), or first diagnosis of AAA (for incidence of AAA), or first diagnosis of CHD (for incidence of CHD). We obtained the information about first diagnosis of AAA/CHD by linking to Swedish national registers (the Swedish Inpatient Register, the hospital-based outpatient register, and the Cause of Death Register) using the 10-digit personal identification number which is unique to each Swedish resident.^15^ The Inpatient Register includes information on dates of admission and discharge as well as diagnostic and procedural codes from all hospitalizations in Sweden. The inpatient register has been operating in south of Sweden since 1970 and has complete national coverage since 1987. The hospital-based outpatient register has been operating with national coverage since 2001. Information on vital status and emigration was provided by the Swedish population register. The diagnoses are coded using a Swedish revision of the *International Classification of Disease* (*ICD*).^16^ The ninth edition (*ICD-9*) was used between 1987 and 1996, and the 10th edition (*ICD-10*) has been used since 1997. Incident AAA was defined based on *ICD-9* code 441D-441E and *ICD-10* code I713-I716 or death attributable to AAA. Coronary heart disease was defined based on *ICD-9* code 410 and *ICD-10* code I21 or death attributable to CHD (*ICD-9* code 410-414; *ICD-10* code I20-25). Moreover, AAA was classified as nonsevere and severe AAA (non-sAAA and sAAA, respectively), based on information in the registers. Severe AAA was defined as (1) an AAA as underlying or primary cause of death, or (2) surgery for AAA within 60 days after first diagnosis, or (3) ruptured AAA.

A validation of the diagnoses or surgical procedures was performed, by review of records for 100 patients with a diagnosis of AAA from the uptake area of Skåne University hospital in Malmö. Between January 1, 2016, and December 31, 2016, 173 patients were diagnosed with AAA or ruptured AAA. Eighty-two patients with diagnosis of AAA and 18 patients with ruptured AAA were randomly selected for the validation procedure using patient record data. The confirmed diagnoses, emergency operation rate, sources of diagnosis, and mortality were summarized in Supplementary Table 1 (modified from Bergwall et al).^17^

### Statistical Analyses

We presented the baseline information in Table 1 by dividing participants into 4 groups: (1) participants who developed neither AAA nor CHD during follow-up; (2) participants who developed AAA, but not CHD, during follow-up; (3) participants who developed CHD, but not AAA, during follow-up; and (4) participants who developed both AAA and CHD during follow-up. Because the distribution of data on ApoA1, ApoB, total leukocyte count, neutrophil count, lymphocyte count, and mixed cell count were skewed, natural logarithm transformations were applied to normalize the data. Analysis of variance was used for continuous variables and χ^2^ test for categorical variables, to compare differences in baseline characteristics across the 4 groups as well as between groups 2 and 3. For each set of analyses, 2 separate analyses were conducted using either incident AAA or incident CHD as the outcome. Cox regression with time-to-event as timescale was used to estimate hazard ratios (HRs) and 95% CIs for incident AAA or CHD. We adjusted for age and sex in model 1, and age, sex, marital status, education level, smoking status, DM, use of antihypertensive, lipid-lowing medications, waist circumference, systolic BP (SBP), ApoB/ApoA1 ratio, and total leukocyte count in model 2.

**Table 1.** General Characteristics According to Incidence of AAA and CHD During Follow-Up.^a,b,c^

MDCS, n = 26 688	Group 1 without AAA or CHD, n = 23 221	Group 2 incident AAA alone, n = 338	Group 3 incident CHD alone, n = 3020	Group 4 incident AAA and CHD, n = 109	*P*
Age (years)	57.5 ± 7.6	61.1 ± 6.7	61.5 ± 7.0	59.9 ± 6.8	<.001
Sex, n (%)					<.001
Male	8194 (35.3)	246 (72.8)^d^	1734 (57.4)^d^	88 (80.7)	
Female	15 027 (64.7)	92 (27.2)	1286 (42.6)	21 (19.3)	
Marriage status, n (%)					.002
Married	15 130 (65.2)	238 (70.4)^d^	1952 (64.6)^d^	83 (76.1)	
Single	2242 (9.7)	17 (5.0)	289 (9.6)	10 (9.2)	
Divorced	4162 (17.9)	64 (18.9)	517 (17.1)	12 (11.0)	
Widow/widower	1687 (7.3)	19 (5.6)	262 (8.7)	4 (3.7)	
Educational level, n (%)					<.001
Low	9285 (40.0)	188 (55.6)	1582 (52.4)	55 (50.5)	
Median	6185 (26.6)	70 (20.7)	720 (23.8)	22 (20.2)	
High	7751 (33.4)	80 (23.7)	718 (23.8)	32 (29.4)	
Smoking, n (%)					<.001
Regularly	5252 (22.6)	178 (52.7)^d^	862 (28.5)^d^	67 (61.5)	
Occasionally	1041 (4.5)	21 (6.2)	138 (4.6)	5 (4.6)	
Formerly	7713 (33.2)	100 (29.6)	1050 (34.8)	28 (25.7)	
Never	9215 (39.7)	39 (11.5)	970 (32.1)	9 (8.3)	
History of diabetes, n (%)	786 (3.4)	11 (3.2)^d^	295 (9.8)^d^	7 (6.4)	<.001
Antihypertensive medication, n (%)	3565 (15.4)	89 (26.3)	829 (27.5)	34 (31.2)	<.001
Antilipid medication, n (%)	471 (2.0)	23 (6.8)	148 (4.9)	9 (8.3)	<.001
Waist circumference (cm)	83.1 ± 14.6	90.8 ± 12.4	89.2 ± 12.6	93.5 ± 11.6	<.001
Systolic blood pressure (mm Hg)	139.9 ± 19.6	147.0 ± 19.9^d^	149.3 ± 20.4^d^	152.1 ± 20.4	<.001
Diastolic blood pressure (mm Hg)	85.1 ± 9.9	89.9 ± 9.6^d^	88.2 ± 10.0^d^	92.1 ± 10.1	<.001
ApoA1 (mg/dL)	156 (139, 175)	139 (125, 156)^d^	147 (131, 165)^d^	132 (122, 151.5)	<.001
ApoB (mg/dL)	104 (88, 121)	118 (103, 134)^d^	114 (99, 132)^d^	124 (107, 139)	<.001
ApoB/ApoA1 ratio	0.66 (0.53, 0.81)	0.83 (0.70, 0.99)^d^	0.78 (0.64, 0.94)^d^	0.91 (0.75, 1.08)	<.001
Total leukocyte count (10^9^ /L)	6.0 (5.2, 7.2)	7.0 (5.7, 8.2)^d^	6.4 (5.4, 7.6)^d^	7.1 (6.0, 8.6)	<.001
Neutrophil count (10^9^ /L)	3.6 (3.0, 4.5)	4.2 (3.4, 5.3)^d^	3.9 (3.2, 4.9)^d^	4.5 (3.5, 5.5)	<.001
Lymphocyte count (10^9^ /L)	1.9 (1.5, 2.3)	2 (1.6, 2.4)^d^	1.9 (1.5, 2.3)^d^	2.1 (1.8, 2.6)	<.001
Mixed cell count (10^9^ L)	0.5 (0.4, 0.6)	0.5 (0.4, 0.7)	0.5 (0.4, 0.7)	0.6 (0.4, 0.7)	<.001
NLR	2.0 (1.5, 2.5)	2.1 (1.7, 2.7)	2.1 (1.6, 2.6)	2.1 (1.7, 2.6)	<.001
Deaths during follow-up, n (%)	6967 (30.0)	194 (57.4)	1828 (60.5)	71 (65.1)	<.001

Abbreviations: AAA, abdominal aortic aneurysm; ApoA1, apolipoproteins A1; ApoB, apolipoproteins B; CHD, coronary heart disease; MDCS, Malmö Diet and Cancer study; NLR, neutrophil to lymphocyte ratio.

^a^ Continuous variables were shown as mean ± standard deviation or median (Q25, Q75); categorical variables were shown as number (%).

^b^ Natural logarithm transformation was applied for variables with non-normal distribution.

^c^ Analysis of variance was used for continuous variables and χ^2^ test used for categorical variables.

^d^ Significant difference was found when participants with incidence AAA alone were compared with those with CHD alone.

We further investigated whether the observed association of factors with each outcome (AAA and CHD) in the separate analyses was similar. For this purpose, we used a competing risks approach described by Lunn and McNeil.^14,18,19^ Briefly, the data set was duplicated with 2 rows per individual and was stratified on these rows. The outcomes of AAA and CHD were separated into these strata. Unlike the original method, in this study, individuals were included in both strata if they developed both AAA and CHD during follow-up. An analysis was then conducted with duplicated covariates so that each covariate was allowed to have different effects in each stratum. The HRs (95% CIs) derived from this analysis are identical to those obtained from separate Cox models fitted in the original data set. Another analysis was then conducted, with one variable unduplicated. This forces the effect measure for this variable to be the same for both strata. This analysis is then compared to the one with differential effects using the likelihood ratio test, with 1 degree of freedom, in order to derive a *P* value for the difference in effect measures for the unduplicated variate. These steps were repeated for each variable in multivariate model. We also carried out secondary analyses excluding those who developed both AAA and CHD. Furthermore, we compared the risk factors between sAAA and non-sAAA and between sAAA and CHD. Finally, we stratified the study population by sex and compared the risk factors between AAA and CHD.

All analyses were performed using the Statistical Analysis System version 9.4 for Windows (SAS Institute Inc). A 2-tailed *P* < .05 was considered significant.

## Results

### Baseline Characteristics

In MDCS (n = 26 688), a total of 447 and 3129 individuals developed AAA (mean follow-up of 19.8 ± 5.4 years) and CHD (mean follow-up of 19.3 ± 5.9 years), respectively. Among them, 109 individuals had both AAA and CHD. Incidence rates for all patients and for subgroups of risk factors are presented in Supplement Table 2. Baseline characteristics of participants according to AAA and CHD event status during follow-up are presented in Table 1. Compared with those who only developed CHD (group 3), individuals with AAA only (group 2) had higher diastolic BP (DBP), ApoB level, ApoB/ApoA1 ratio, total leukocyte count, neutrophil count, and lymphocyte count, but lower SBP and ApoA1 level. In addition, a larger proportion of patients with AAA were male, married, and smokers, whereas the proportion with DM was smaller.

### Risk Profiles for Prediction of Incident AAA and CHD

The age- and sex-adjusted and multivariate-adjusted HRs for incident AAA and CHD are summarized in Table 2. After adjustment for other risk factors, smoking, antihypertensive medications, lipid-lowing medications, SBP/DBP, ApoA1 (negative), ApoB, ApoB/ApoA1 ratio, total leukocyte count, neutrophil count, and neutrophil to lymphocyte ratio were associated with an increased risk for both AAA and CHD. However, some factors were associated with CHD, but not with AAA, including being married (HR_CHD_ = 0.85, 95% CI, 0.78-0.91; HR_AAA_ = 1.17, 95% CI, 0.95-1.44), educational level (low vs high: HR_CHD_ = 1.25, 95% CI, 1.14-1.37; HR_AAA_ = 1.15, 95% CI, 0.92-1.45), increased waist circumference (HR_CHD_ = 1.09, 95% CI, 1.04-1.14; HR_AAA_ = 1.06, 95% CI, 0.94-1.2), and DM (HR_CHD_ = 1.99, 95% CI, 1.76-2.25; HR_AAA_ = 0.75, 95% CI, 0.46-1.2). Patterns of risk factors for AAA and CHD were generally consistent in secondary analyses excluding those who developed both AAA and CHD (Supplementary Table 3). A stratified analysis of men and women is presented in Supplementary Figure 2.

**Table 2.** Comparison of Risk Factors for AAA and CHD.^a,b,c^

		AAA, n = 447			CHD, n = 3129			*P* value for equal associations^d^
MDCS, n = 26 688		Incident	HR (95% CI)	*P*	Incident	HR (95% CI)	*P*	
Married	Model 1	447	0.97 (0.79-1.19)	.768	3129	0.79 (0.74-0.85)	<.001	.073
	Model 2	447	1.17 (0.95-1.44)	.152	3129	0.85 (0.78-0.91)	<.001	.004
Educational level	Model 1	447			3129			.345
High			Reference			Reference		
Low			1.57 (1.25-1.97)	<.001		1.48 (1.36-1.62)	<.001	
Median			1.21 (0.91-1.59)	.186		1.28 (1.16-1.42)	<.001	
	Model 2	447			3129			.242
High			Reference			Reference		
Low			1.15 (0.92-1.45)	.219		1.25 (1.14-1.37)	<.001	
Median			0.98 (0.74-1.30)	.895		1.17 (1.06-1.30)	.003	
Smokers	Model 1	447			3129			<.001
No, never			Reference			Reference		
Yes, regularly			10.71 (7.83-14.64)	<.001		2.07 (1.89-2.27)	<.001	
Yes, occasionally			5.37 (3.32-8.68)	<.001		1.56 (1.31-1.86)	<.001	
Yes, formerly			2.48 (1.77-3.46)	<.001		1.18 (1.08-1.29)	<.001	
	Model 2	447			3129			<.001
No, never			Reference			Reference		
Yes, regularly			9.03 (6.49-12.57)	<.001		1.85 (1.68-2.04)	<.001	
Yes, occasionally			5.16 (3.18-8.36)	<.001		1.51 (1.27-1.81)	<.001	
Yes, formerly			2.35 (1.68-3.29)	<.001		1.14 (1.04-1.24)	.005	
History of diabetes	Model 1	447	0.93 (0.58-1.49)	.754	3129	2.50 (2.22-2.82)	<.001	<.001
	Model 2	447	0.75 (0.46-1.20)	.227	3129	1.99 (1.76-2.25)	<.001	<.001
Antihypertensive medication	Model 1	447	1.72 (1.40-2.13)	<.001	3129	1.68 (1.55-1.82)	<.001	.825
	Model 2	447	1.52 (1.22-1.91)	<.001	3129	1.30 (1.20-1.42)	<.001	.203
Antilipid medication	Model 1	447	2.34 (1.63-3.36)	<.001	3129	1.71 (1.46-2.01)	<.001	.135
	Model 2	447	1.98 (1.37-2.86)	<.001	3129	1.42 (1.21-1.68)	<.001	.119
Waist circumference	Model 1	447	1.24 (1.10-1.39)	.001	3129	1.29 (1.23-1.34)	<.001	.542
	Model 2	447	1.06 (0.94-1.20)	.366	3129	1.09 (1.04-1.14)	<.001	.674
Systolic blood pressure	Model 1	447	1.13 (1.07-1.18)	<.001	3129	1.15 (1.13-1.17)	<.001	.434
	Model 2	447	1.11 (1.05-1.16)	<.001	3129	1.12 (1.10-1.14)	<.001	.666
Diastolic blood pressure	Model 1	447	1.40 (1.29-1.53)	<.001	3129	1.21 (1.16-1.25)	<.001	.002
	Model 2	447	1.39 (1.27-1.52)	<.001	3129	1.14 (1.10-1.18)	<.001	<.001
ApoA1	Model 1	447	0.09 (0.06-0.15)	<.001	3129	0.24 (0.20-0.30)	<.001	<.001
	Model 2	447	0.15 (0.09-0.26)	<.001	3129	0.37 (0.30-0.47)	<.001	.003
ApoB	Model 1	447	6.34 (4.16-9.66)	<.001	3129	3.39 (2.91-3.96)	<.001	.006
	Model 2	447	3.54 (2.30-5.46)	<.001	3129	2.34 (2.00-2.74)	<.001	.078
ApoB/ApoA1 ratio	Model 1	447	8.38 (5.94-11.82)	<.001	3129	3.56 (3.14-4.04)	<.001	<.001
	Model 2	447	4.82 (3.38-6.87)	<.001	3129	2.49 (2.19-2.84)	<.001	.001
Total leukocyte count	Model 1	447	8.60 (6.02-12.30)	<.001	3129	2.82 (2.45-3.25)	<.001	<.001
	Model 2	447	1.84 (1.22-2.76)	.004	3129	1.43 (1.22-1.67)	<.001	.257
Neutrophil count	Model 1	447	4.90 (3.68-6.51)	<.001	3129	2.13 (1.91-2.38)	<.001	<.001
	Model 2	447	1.72 (1.25-2.35)	<.001	3129	1.38 (1.22-1.55)	<.001	.198
Lymphocyte count	Model 1	447	3.07 (2.29-4.11)	<.001	3129	1.63 (1.45-1.83)	<.001	<.001
	Model 2	447	1.12 (0.80-1.56)	.506	3129	0.99 (0.87-1.12)	.839	.488
Mixed cell count	Model 1	447	1.60 (1.25-2.04)	<.001	3129	1.23 (1.13-1.34)	<.001	.045
	Model 2	447	1.04 (0.86-1.26)	.667	3129	1.02 (0.94-1.10)	.666	.808
NLR	Model 1	447	1.48 (1.17-1.89)	.001	3129	1.24 (1.13-1.36)	<.001	.180
	Model 2	447	1.33 (1.03-1.71)	.028	3129	1.23 (1.12-1.35)	<.001	.583

Abbreviations: AAA, abdominal aortic aneurysm; ApoA1, apolipoproteins A1; ApoB, apolipoproteins B; CHD, coronary heart disease; HR, hazard ratio; MDCS, Malmö Diet and Cancer study; NLR, neutrophil to lymphocyte ratio.

^a^ Model 1: adjusted for age and sex.

^b^ Model 2: adjusted for age, sex, marriage status, education, smoking, diabetes, waist, systolic blood pressure, antihypertensive medication, ApoB/ApoA1 ratio, antilipid medication, and white blood cell count. ApoA1 and ApoB are not adjusted for ApoB/ApoA1 ratio. Diastolic blood pressure is not adjusted for systolic blood pressure. Differentiated leucocyte not adjusted for white blood cell count.

^c^ Diastolic blood pressure and systolic blood pressures were grouped by 10 mm Hg intervals; waist circumference was grouped by quartile.

^d^ A significant *P* value indicates that the risk factor has different associations with AAA and CHD, respectively. The Lunn-McNeil method and log likelihood test were used to calculate the *P* values (see section “Methods” for details).

### Comparisons of Risk Profiles for Incident AAA and CHD

**Figure 1.**
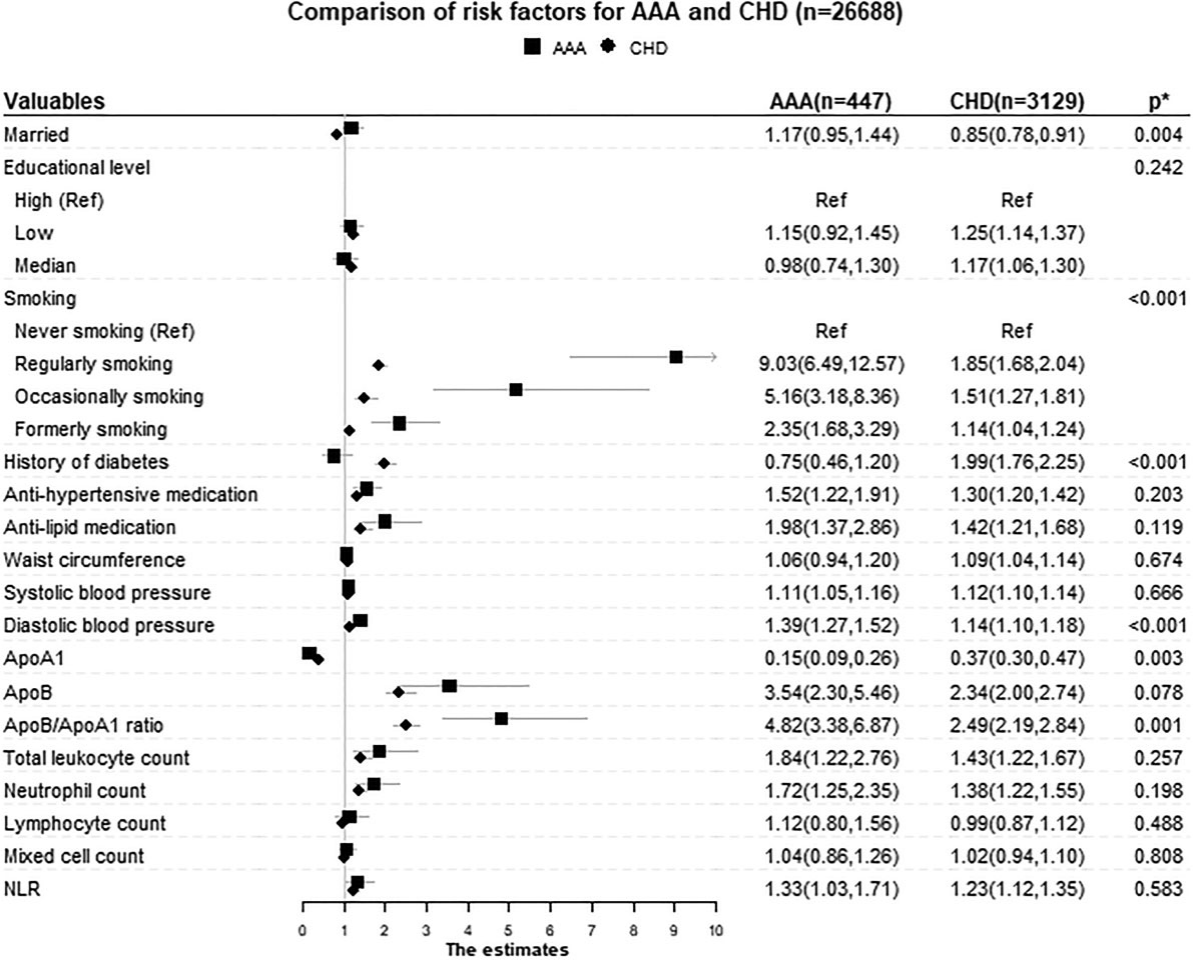
Comparisons of different risk factors for AAA and CHD. The model was adjusted for age, sex, marriage status, education, smoking, diabetes, waist, systolic blood pressure, antihypertensive medication, ApoB/ApoA1 ratio, antilipid medication, and white blood cell count. ApoA1 and ApoB are not adjusted for ApoB/ApoA1 ratio. Diastolic blood pressure was not adjusted for systolic blood pressure. The differentiated leucocyte count was not adjusted for white blood cell count. * *P* value for equal associations. A significant *P* value indicates that the risk factor has different associations with AAA and CHD, respectively. The Lunn-McNeil method and log likelihood test were used to calculate the *P* values (see section “Methods” for details). AAA indicates abdominal aortic aneurysm; ApoA1, apolipoproteins A1; ApoB, apolipoproteins B; CHD, coronary heart disease; NLR, neutrophil to lymphocyte ratio.

The analyses of differential associations between AAA and CHD outcomes for each factor are presented in Table 2 and Figure 1. After adjusting for other risk factors, the Lunn-McNeil test showed that marital status, smoking, DM, DBP, ApoA1, and ApoB/ApoA1 ratio had significantly different relationships with AAA and CHD, respectively (*P* for equal associations = .004, <.001, <.001, <.001, =.003, and =.001, respectively). The HRs for smoking, DBP, and ApoB/ApoA1 ratio were significantly stronger for AAA than for CHD. Although ApoA1 had protective effects for both AAA and CHD, the HR for AAA was considerably stronger than for CHD. Diabetes mellitus and being unmarried were associated with an increased risk of CHD, but were not associated with AAA.

### Risk Factors for Incident sAAA and Non-sAAA

The risk factors for sAAA and non-sAAA are presented in Supplementary Table 4. After adjusting for other factors, the effects of the risk factors showed no significant difference between the 2 subtypes.

## Discussion

Both AAA and CHD are strongly associated with atherosclerosis. However, since the pathogenesis of both diseases can be regarded as multifactorial, their risk factors could be different.^7,10^ We sought to compare the risk factor profiles for AAA and CHD. Overall, the results indicated substantial similarities in the risk profiles, but there were also important differences. The relationships with smoking, DBP, and ApoB/ApoA1 ratio were considerably stronger for AAA than for CHD. Although ApoA1 had protective effects for both AAA and CHD, the effect for AAA was considerably stronger than for CHD. Additionally, DM and being unmarried were associated with an increased risk of CHD, but not with AAA. Therefore, this study confirmed that although AAA and CHD share many risk factors, the risk factor profiles also show important differences. Studies of risk factors for atherosclerosis in different vascular beds could improve our understanding of the pathogenesis and treatment of atherosclerotic disease.

Clinical studies have shown that among patients with angiography-verified CHD, the prevalence of AAA is high, and the prevalence is higher among patients with more severe CHD.^6,20^ Moreover, studies indicate that AAA and CHD share some genetic pathways and were related to an identical genetic risk score.^8,21^ Although AAA shares some risk factors with CHD, the magnitude and direction of this association are not always consistent. For example, opposite relationships have been reported for atheroma extent and AAA severity, the risk of smoking for AAA was at least 2-fold greater than that for CHD, and DM showed opposite associations with AAA and CHD.^7,10,22,23^ These findings support the view that shared environmental and genetic risk factors may promote the development of both CHD and AAA in some patients, but the mechanisms involved may differ.^8,24^ Besides, the fact that coronary arteries originate from the neural crest whereas the abdominal aorta originates from the mesoderm could make them react differently to harmful stimuli.^25,26^

Patients with DM have 2- to 4-fold increase in the risk of CHD.^27^ Diabetes mellitus adversely influence the functions of endothelial cell, vascular smooth muscle, and platelets, leading to atherosclerotic disease.^27^ However, epidemiological data have suggested that patients with DM have a lower incidence of AAA.^11,23^ A systematic review revealed that the prevalence of DM in patients with AAA was 6% to 14%, whereas in control patients without AAA, prevalence ranged from 17% to 36%.^12^ The full biological explanation for this relationship is still unknown. Animal-based studies exhibited a protective effect of hyperglycemia for AAA, by reduced aortic mural neovascularization, macrophage infiltration, and medial elastolysis.^28^ In vitro, activated monocytes with glycated collagen lattices reduced secretion of matrix metalloproteinases in association with cross-linkage.^29^ In line with these studies, we found that DM would increase the risk for CHD, but not for AAA, and formally tested this difference for significance.

Cigarette smoking is an established risk factor for CHD.^30^ Smoking could increase inflammation, thrombosis, and oxidation of low-density lipoprotein cholesterol (LDL-C), which impacts all phases of CHD from endothelial dysfunction to acute clinical events, the latter being largely thrombotic.^31^ Not only the prevalence of AAA but also the size of aneurysms and the risk of rupture have strong positive associations with the quantity and duration of smoking and inverse associations with the years after smoking cessation.^32,33^ Smoking cessation is also associated with a reduced rate of aneurysmal growth.^34^ Our results supported that AAA was more sensitive to smoking than CHD. It is possible that smoking could disrupt collagen synthesis and alter expression of metalloproteinases, which could increase formation of aneurysms, but the mechanisms need to be explored.^35^

Hypertension is a risk factor for both AAA and CHD.^36,37^ However, the results for DBP has been inconsistent and some studies indicated that DBP, but not SBP, was positively associated with AAA,^38^ while others found that AAA was unrelated to DBP.^39^ In our cohort, both SBP and DBP were risk factors for AAA as well as CHD, but DBP showed a stronger association with the incidence of AAA than with CHD.

The relation between serum lipids and the risk of aortic aneurysms has been inconsistent in observational studies.^32,39,40^ However, evidence from Mendelian randomization studies showed that genetic elevation of LDL-C, high-density lipoprotein cholesterol (HDL-C), and total triglyceride appeared to be independent risk factors for AAA whereas only LDL-C was found to be independently associated with CHD risk.^22,41^ These genetic studies indicate that more lipid pathways were involved in the process of AAA, which may explain why ApoA1 (a component of HDL-C) had a stronger protective effect to AAA than CHD in the present study.

### Limitations and Strengths

The large sample size and long-term follow-up are a major strength of the study. Outcome data were retrieved from registers with national coverage, which helped eliminate recall bias. The ultrasound AAA screening of 65 years old men started in our area in 2010, which potentially could lead to a larger proportion of smaller AAA (non-sAAA) being detected after this date. However, only a minor proportion of the men in MDCS were born after 1945 and eligible for the screening. Few cases were, therefore, detected by screening. The diagnosis of AAA was further validated to ensure the accuracy, and most cases had relatively large-sized AAA at diagnosis, which indicates a long-term exposure of pathophysiological mechanisms promoting AAA growth (Supplementary Table 1).

Although we had information about smoking and former smoking, we did not have information about pack-years. Both AAA and CHD might be inflammation-driven diseases. Although information about leukocyte and neutrophil counts, a classical marker of inflammation, was available for all individuals, we lack details of other inflammatory biomarkers. And also, there were other factors probably related to risk of both AAA and CHD, information of which however is lacking in our data set, such as estimated glomerular filtration rate.

## Conclusion

The results from this big population study confirm that the risk factor profiles for AAA and CHD showed not only many similarities but also several important differences.

## Supplemental Material

Supp_Mat - Comparisons of Risk Factors for Abdominal Aortic Aneurysm and Coronary Heart Disease: A Prospective Cohort Study Click here for additional data file. Supp_Mat for Comparisons of Risk Factors for Abdominal Aortic Aneurysm and Coronary Heart Disease: A Prospective Cohort Study by Jun Xiao, Yan Borné, Xue Bao, Margaretha Persson, Anders Gottsäter, Stefan Acosta and Gunnar Engström in Angiology
