# Supplementary material for: Comparisons of Risk Factors for Abdominal Aortic Aneurysm and Coronary Heart Disease: A Prospective Cohort Study
Source: Angiology. 2020 Aug 7;72(1):24–31. doi: 10.1177/0003319720946976 (PMC7711307; doi:10.1177/0003319720946976)
Supplement: Supp_Mat - Comparisons of Risk Factors for Abdominal Aortic Aneurysm and Coronary Heart Disease: A Prospective Cohort Study [file Supp_Mat.pdf]

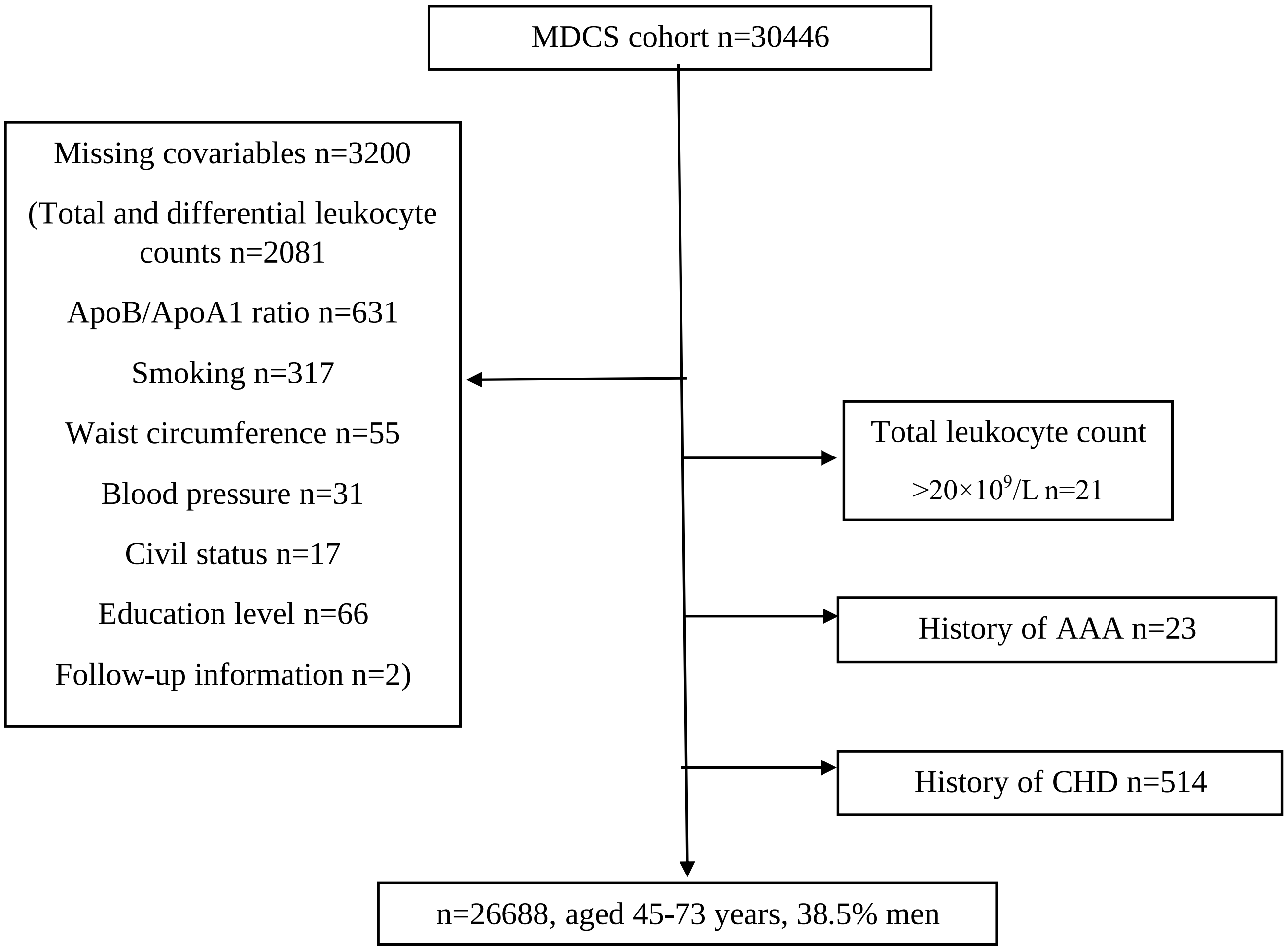

Supplementary Figure 1. Individuals included in this research.

Abbreviations: MDCS, Malmö Diet and Cancer study; ApoA1, apolipoproteins A1; ApoB, apolipoproteins B; AAA, abdominal aortic aneurysm; CHD, coronary heart disease.

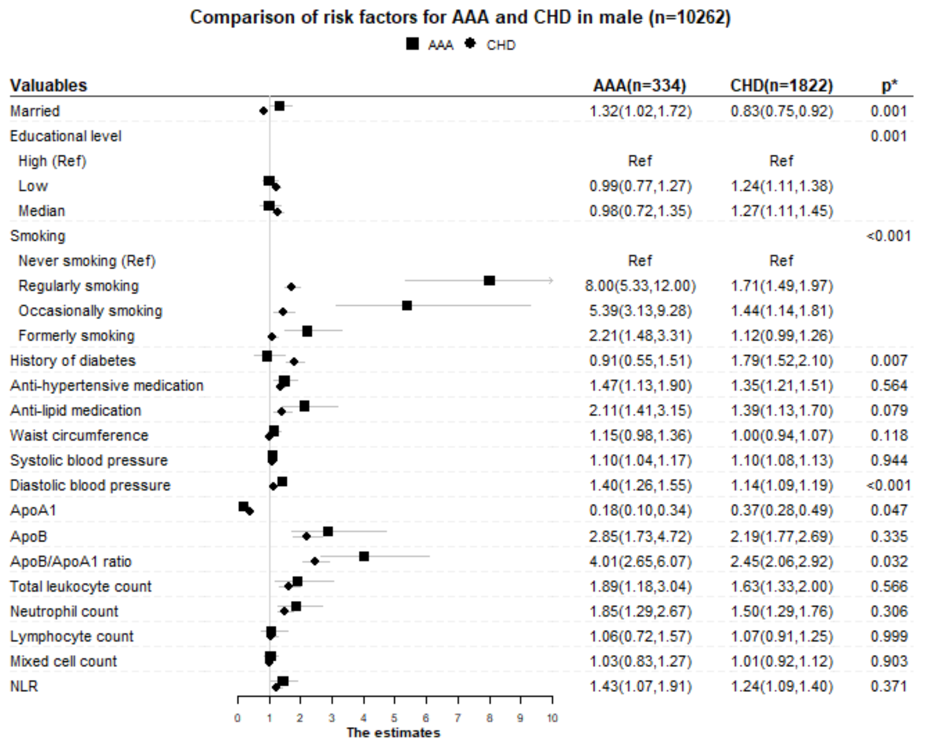

A

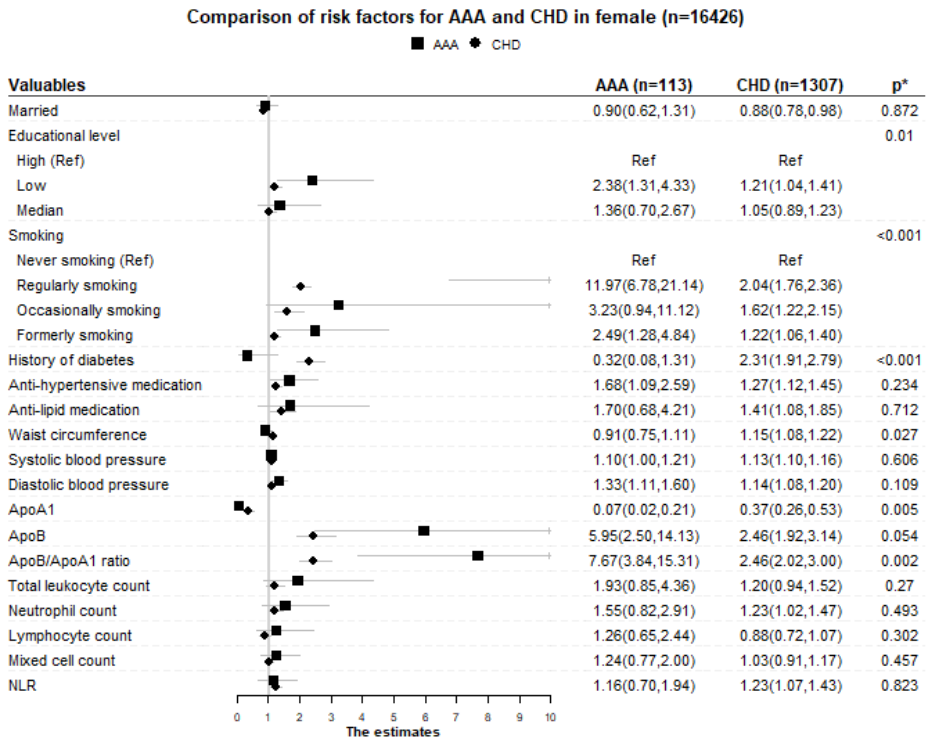

B

Supplementary Figure 2. Comparisons of different risk factors for AAA and CHD in male (A) and female (B) subjects.

The model was adjusted for age, sex, marriage status, education, smoking, diabetes, waist, systolic blood pressure, anti-hypertensive medication, ApoB/ApoA1 ratio, anti-lipid medication, and white blood cell count. ApoA1 and ApoB not adjusted for ApoB/ApoA1 ratio. Diastolic blood pressure not adjusted for systolic blood pressure. Differentiated leucocyte not adjusted for white blood cell count.

*p-value for equal associations. A significant p-value indicates that the risk factor has different associations with AAA and CHD, respectively. The Lunn-McNeil method and log likelihood test were used to calculate the p-values (see methods for details).

Abbreviations: AAA, abdominal aortic aneurysm; CHD, coronary heart disease; ApoA1, apolipoproteins A1; ApoB, apolipoproteins B; NLR, neutrophil to lymphocyte ratio.

Supplementary Table 1. Validation of patients with AAA and ruptured AAA (rAAA).

**Characteristics**	**AAA** (n=80)	**rAAA** (n=18)	p
**Confirmed diagnosis, (%)**	76/80 (95%)	17/18 (94.4)	1.000
**Median (IQR) age; years**	76 (71 – 80)	84 (78 – 85)	<0.001
**Male sex, (%)**	58/76 (76.3)	12/17 (70.6)	0.620
**Median maximal AAA diameter (IQR); mm**	50 (40 – 64)	78 (56 – 98)	<0.001
**Operated at the time of diagnosis, (%)**	5/76 (6.6)	10/17 (58.8)	
EVAR	5	8	<0.001
Open repair	0	2	
**Mortality within 30 days of diagnosis, (%)**	0/76 (0)	11/17 (64.7)	<0.001
**Initial mode of diagnosis, (%)**			
Imaging due to AAA-related symptoms	2	16	
Accidental finding at any imaging	56	0	
Palpation	12	0	
Autopsy	0	1	
Organized ultrasound screening for AAA	2	0	
Non-organized ultrasound screening for AAA	4	0	

In our study population, 447 individuals developed AAA. Among them, 212 (47.4%) were from the inpatient register, 211(47.2%) from hospital-based outpatient care and 24 (5.4%) from the cause-of-death register. Among the 134 individuals with severe AAA, 88(65.7%) from the inpatient

register, 26(19.4%) from hospital-based outpatient care and 20 (14.9%) from the cause-of-death register. Corresponding number in the 313 non-severe AAA population were 124 (39.6%), 185 (59.1%) and 4 (1.1%), respectively. There were 70 (15.7%) ruptured AAA patients in this study. Among them, 43 (61.4%) were from the inpatient register, 7 (10%) from hospital-based outpatient care and 20 (28.6%) from the cause-of-death register; the distribution in 373 non-ruptured AAA were 165 (44.2%), 204 (54.7%) and 4 (1.1%), respectively.

Abbreviations: AAA, abdominal aortic aneurysm; rAAA; ruptured abdominal aortic aneurysm, EVAR; Endovascular aneurysm repair, IQR; interquartile range.

Supplementary Table 2. Incidence rates (per 1000 person years) for AAA and CHD in categories of selected risk factors.

n=26688	AAA	CHD
**Exposures**	**Number of cases (per 1000 person-years)**	**Number of cases (per 1000 person-years)**
**All**	447 (0.85)	3129 (6.08)
**Men**	334 (1.72)	1822 (9.79)
**Women**	113 (0.34)	1307 (3.98)
**Smokers**	271 (1.90)	1072 (7.77)
**Diabetes**	18 (0.95)	302 (17.2)
**SBP ≥160 mmHg**	131 (1.33)	1033 (11.02)
**DBP ≥100 mmHg**	93 (1.86)	501 (9.56)
**High ApoA1**	47 (0.12)	523 (3.84)
**High ApoB/ApoA1 ratio**	235 (1.86)	1263 (10.52)

High ApoA1 and high ApoB/ApoA1 were defined as at the 4th quartile of ApoA1 (>174 mg/L) and 4th quartile of ApoB/ApoA1 ratio (>0.834) in this study, respectively. The incident rate was present as per 1000 person-years.

Abbreviations: AAA, abdominal aortic aneurysm; CHD, coronary heart disease; SBP, systolic blood pressure; DBP, diastolic blood pressure; ApoA1, apolipoproteins A1; ApoB, apolipoproteins B.

Supplementary Table 3. Comparison of risk factors for AAA and CHD (excluding those who developed both AAA and CHD).

**MDCS (n=26579)**		**AAA (n=338)**			**CHD (n=3020)**			p-value for equal associations*****
		**Incident**	**HR (95%CI)**	p	**Incident**	**HR (95%CI)**	p	
**Married**	Model 1	338	0.91(0.72,1.16)	0.448	3020	0.78(0.73,0.85)	<0.001	0.227
	Model 2	338	1.1(0.86,1.39)	0.457	3020	0.83(0.77,0.9)	<0.001	**0.030**
**Educational level**	Model 1	338			3020			0.266
High			ref			ref		
Low			1.67(1.28,2.18)	<0.001		1.48(1.36,1.62)	<0.001	
Median			1.26(0.91,1.74)	0.159		1.29(1.16,1.43)	<0.001	
	Model 2	338			3020			0.411
High			ref			ref		
Low			1.27(0.98,1.66)	0.075		1.26(1.15,1.38)	<0.001	
Median			1.06(0.77,1.47)	0.724		1.18(1.06,1.31)	0.002	
**Smoking**	Model 1	338			3020			<0.001
No, never			ref			ref		
Yes, regularly			9.95(7.01,14.13)	<0.001		1.97(1.79,2.16)	<0.001	
Yes, occasionally			5.54(3.25,9.45)	<0.001		1.53(1.28,1.84)	<0.001	
Yes, formerly			2.44(1.68,3.55)	<0.001		1.17(1.07,1.28)	.001	

	Model 2	338			3020			**<0.001**
No, never			ref			ref		
Yes, regularly			8.52(5.89,12.35)	<0.001		1.77(1.6,1.96)	<0.001	
Yes, occasionally			5.35(3.13,9.15)	<0.001		1.49(1.24,1.78)	<0.001	
Yes, formerly			2.34(1.61,3.41)	<0.001		1.13(1.03,1.24)	0.008	
**History of diabetes**	Model 1	338	0.74(0.41,1.35)	0.328	3020	2.54(2.25,2.87)	<0.001	<0.001
	Model 2	338	0.61(0.33,1.12)	0.112	3020	2.03(1.8,2.3)	<0.001	**<0.001**
**Anti-hypertensive medication**	Model 1	338	1.6(1.25,2.04)	<0.001	3020	1.67(1.54,1.81)	<0.001	0.726
	Model 2	338	1.5(1.16,1.95)	0.002	3020	1.3(1.2,1.42)	<0.001	0.312
**Anti-lipid medication**	Model 1	338	2.21(1.44,3.38)	<0.001	3020	1.68(1.42,1.98)	<0.001	0.249
	Model 2	338	1.9(1.23,2.92)	0.004	3020	1.39(1.18,1.65)	<0.001	0.207
**Waist circumference**	Model 1	338	1.19(1.04,1.36)	0.010	3020	1.28(1.23,1.34)	<0.001	0.309
	Model 2	338	1.05(0.91,1.21)	0.512	3020	1.09(1.04,1.14)	<0.001	0.609
**Systolic blood pressure**	Model 1	338	1.08(1.02,1.15)	0.005	3020	1.15(1.13,1.17)	<0.001	0.058
	Model 2	338	1.07(1.01,1.13)	0.027	3020	1.11(1.09,1.14)	<0.001	0.182
**Diastolic blood pressure**	Model 1	338	1.34(1.21,1.49)	<0.001	3020	1.19(1.15,1.24)	<0.001	0.035
	Model 2	338	1.34(1.21,1.49)	<0.001	3020	1.13(1.09,1.17)	<0.001	**0.002**
**ApoA1**	Model 1	338	0.11(0.06,0.2)	<0.001	3020	0.26(0.21,0.32)	<0.001	0.010
	Model 2	338	0.18(0.1,0.34)	<0.001	3020	0.39(0.31,0.49)	<0.001	**0.024**

**ApoB**	Model 1	338	5.25(3.24,8.52)	<0.001	3020	3.26(2.78,3.82)	<0.001	0.065
	Model 2	338	3.14(1.91,5.17)	<0.001	3020	2.28(1.94,2.67)	<0.001	0.224
**ApoB/ApoA1 ratio**	Model 1	338	6.74(4.54,10.01)	<0.001	3020	3.4(2.99,3.86)	<0.001	0.001
	Model 2	338	4.11(2.73,6.17)	<0.001	3020	2.4(2.1,2.74)	<0.001	**<0.001**
**Total leukocyte count**	Model 1	338	7.19(4.75,10.9)	<0.001	3020	2.66(2.31,3.08)	<0.001	<0.001
	Model 2	338	1.68(1.05,2.69)	0.030	3020	1.4(1.19,1.64)	<0.001	0.462
**Neutrophil count**	Model 1	338	4.42(3.18,6.16)	<0.001	3020	2.05(1.83,2.29)	<0.001	<0.001
	Model 2	338	1.67(1.16,2.4)	0.006	3020	1.36(1.2,1.53)	<0.001	0.290
**Lymphocyte count**	Model 1	338	2.63(1.87,3.7)	<0.001	3020	1.56(1.39,1.76)	<0.001	0.006
	Model 2	338	1.01(0.69,1.48)	0.948	3020	0.97(0.86,1.1)	0.646	0.836
**Mixed cell count**	Model 1	338	1.61(1.22,2.13)	0.001	3020	1.23(1.13,1.34)	<0.001	0.065
	Model 2	338	1.07(0.85,1.34)	0.592	3020	1.02(0.94,1.1)	0.606	0.729
**NLR**	Model 1	338	1.52(1.15,2.01)	0.003	3020	1.24(1.13,1.36)	<0.001	0.174
	Model 2	338	1.38(1.03,1.84)	0.029	3020	1.23(1.12,1.36)	<0.001	0.470

Model 1: adjusted for age and sex

Model 2: adjusted for age, sex, marriage status, education, smoking, diabetes, waist, systolic blood pressure, anti-hypertensive medication, ApoB/ApoA1 ratio, anti-lipid medication, and white blood cell count. ApoA1 and ApoB not adjusted for ApoB/ApoA1 ratio. Diastolic blood pressure not adjusted for systolic blood pressure. Differentiated leucocyte not adjusted for white blood cell count.

Diastolic blood pressure and systolic blood pressures were grouped by 10mmHg intervals; Waist circumference was grouped by quartile.

* A significant p-value indicates that the risk factor has different associations with AAA and CHD, respectively. The Lunn-McNeil method and log likelihood test were used to calculate the p-values (see methods for details).

Abbreviations: MDCS, Malmö Diet and Cancer study; AAA, abdominal aortic aneurysm; CHD, coronary heart disease; ApoA1, apolipoproteins A1; ApoB, apolipoproteins B; NLR, neutrophil to lymphocyte ratio.

Supplementary table 4. Comparison of risk factors for sAAA and non-sAAA.

MDCS (n=26688)		sAAA			Non-sAAA			p-value for equal associations *
		**Incident**	**HR (95%CI)**	p	**Incident**	**HR (95%CI)**	p	
**Married**	Model 1	134	1.09(0.73,1.61)	0.675	313	0.92(0.72,1.18)	0.519	0.483
	Model 2	134	1.32(0.89,1.95)	0.175	313	1.1(0.86,1.41)	0.455	0.448
**Educational level**	Model 1	134			313			0.772
High			ref			ref		
Low			1.65(1.09,2.49)	0.018		1.54(1.17,2.01)	0.002	
Median			1.26(0.76,2.11)	0.371		1.18(0.85,1.65)	0.318	
	Model 2	134			313			0.818
High			ref			ref		
Low			1.2(0.79,1.82)	0.395		1.14(0.87,1.49)	0.360	
Median			1.01(0.61,1.69)	0.966		0.98(0.7,1.36)	0.883	
**Smoking**	Model 1	134			313			0.280
No, never			ref			ref		
Yes, regularly			11.97(6.48,22.13)	<.001		10.14(7.05,14.58)	<.001	
Yes, occasionally			7.69(3.3,17.89)	<.001		4.5(2.49,8.14)	<.001	
Yes, formerly			2.66(1.38,5.12)	0.004		2.42(1.64,3.57)	<.001	

	Model 2	134			313			0.312
No, never			ref			ref		
Yes, regularly			9.51(4.99,18.12)	<.001		8.71(5.93,12.8)	<.001	
Yes, occasionally			7.1(3.04,16.62)	<.001		4.39(2.42,7.97)	<.001	
Yes, formerly			2.43(1.26,4.7)	0.008		2.33(1.57,3.44)	<.001	
**History of diabetes**	Model 1	134	1.17(0.55,2.51)	0.689	313	0.82(0.45,1.5)	0.514	0.479
	Model 2	134	0.91(0.42,1.97)	0.808	313	0.66(0.36,1.21)	0.180	0.529
**Anti-hypertensive medication**	Model 1	134	1.9(1.3,2.77)	0.001	313	1.64(1.27,2.12)	<.001	0.540
	Model 2	134	1.59(1.06,2.37)	0.025	313	1.48(1.14,1.94)	0.004	0.790
**Anti-lipid medication**	Model 1	134	3.42(1.96,5.98)	<.001	313	1.85(1.14,2.98)	0.012	0.105
	Model 2	134	2.72(1.54,4.83)	0.001	313	1.59(0.98,2.58)	0.062	0.163
**Waist circumference**	Model 1	134	1.28(1.02,1.59)	0.032	313	1.22(1.06,1.4)	0.005	0.739
	Model 2	134	1.11(0.88,1.4)	0.389	313	1.04(0.9,1.2)	0.601	0.652
**Systolic blood pressure**	Model 1	134	1.12(1.02,1.22)	0.014	313	1.13(1.07,1.2)	<.001	0.821
	Model 2	134	1.08(0.99,1.19)	0.086	313	1.11(1.05,1.18)	0.001	0.668
**Diastolic blood pressure**	Model 1	134	1.5(1.28,1.76)	<.001	313	1.36(1.22,1.51)	<.001	0.325
	Model 2	134	1.47(1.25,1.73)	<.001	313	1.35(1.21,1.5)	<.001	0.384
**ApoA1**	Model 1	134	0.15(0.06,0.4)	<.001	313	0.08(0.04,0.14)	<.001	0.224
	Model 2	134	0.3(0.11,0.83)	0.021	313	0.12(0.07,0.22)	<.001	0.127

**ApoB**	Model 1	134	6.69(3.09,14.49)	<.001	313	6.13(3.71,10.14)	<.001	0.854
	Model 2	134	3.56(1.61,7.88)	0.002	313	3.53(2.11,5.93)	<.001	0.975
**ApoB/ApoA1 ratio**	Model 1	134	6.99(3.73,13.1)	<.001	313	8.94(5.92,13.5)	<.001	0.519
	Model 2	134	3.62(1.91,6.88)	<.001	313	5.4(3.53,8.28)	<.001	0.309
**Total leukocyte count**	Model 1	134	11.09(5.83,21.1)	<.001	313	7.57(4.92,11.63)	<.001	0.333
	Model 2	134	2.52(1.2,5.3)	0.015	313	1.59(0.97,2.58)	0.065	0.308
**Neutrophil count**	Model 1	134	5.33(3.18,8.95)	<.001	313	4.66(3.31,6.56)	<.001	0.673
	Model 2	134	1.86(1.05,3.3)	0.034	313	1.64(1.13,2.39)	0.010	0.725
**Lymphocyte count**	Model 1	134	4.07(2.46,6.75)	<.001	313	2.67(1.87,3.82)	<.001	0.186
	Model 2	134	1.66(0.91,3.02)	0.102	313	0.94(0.63,1.4)	0.766	0.127
**Mixed cell count**	Model 1	134	2.12(1.34,3.35)	0.001	313	1.42(1.07,1.89)	0.015	0.145
	Model 2	134	1.26(0.83,1.9)	0.282	313	0.98(0.79,1.21)	0.860	0.284
**NLR**	Model 1	134	1.28(0.82,2)	0.275	313	1.58(1.18,2.1)	0.002	0.440
	Model 2	134	1.11(0.7,1.77)	0.647	313	1.43(1.06,1.93)	0.021	0.381

Model 1: adjusted for age and sex

Model 2: adjusted for age, sex, marriage status, education, smoking, diabetes, waist, systolic blood pressure, anti-hypertensive medication, ApoB/ApoA1 ratio, anti-lipid medication and white blood cell count. ApoA1 and ApoB not adjusted for ApoB/ApoA1 ratio. Diastolic blood pressure not adjusted for systolic blood pressure. Differentiated leucocyte not adjusted for white blood cell count.

Diastolic blood pressure and systolic blood pressures were grouped by 10 mmHg intervals; Waist circumference was grouped by quartile.

* A significant p-value indicates that the risk factor has different associations with sAAA and non-sAAA, respectively. The Lunn-McNeil method and log likelihood test were used to calculate the p-values (see methods for details).

Abbreviations: MDCS, Malmö Diet and Cancer study; sAAA, severe AAA; ApoA1, apolipoproteins A1; ApoB, apolipoproteins B; NLR, neutrophil to lymphocyte ratio. Severe AAA was defined as a (1) AAA as underlying or primary cause of death, or (2) surgery for AAA within 60 days after first diagnosis, or (3) ruptured AAA.
